# QTL mapping of oat crown rust resistance in Australian fields and identification of a seedling resistance locus in oat line GS7

**DOI:** 10.1007/s00122-025-05145-x

**Published:** 2026-01-19

**Authors:** Duong T. Nguyen, David Lewis, Eva C. Henningsen, Zhouyang Su, Rohit Mago, Jana Sperschneider, Peter N. Dodds, Allan Rattey, Belayneh A. Yimer, Kathy Esvelt Klos, Melania Figueroa

**Affiliations:** 1 Commonwealth Scientific and Industrial Research Organisation, Agriculture and Food, Canberra, ACT 2600 Australia; 2 Commonwealth Scientific and Industrial Research Organisation, Agriculture and Food, Adelaide, SA 5064 Australia; 3 Intergrain, 19 Ambitious Link, Bibra Lake, Perth, WA 6163 Australia; 4 Small Grains and Potato Germplasm Research Unit, USDA-ARS, 1691 South 2700 West, Aberdeen, ID 83210 USA

## Abstract

The development of oat cultivars with resistance to crown rust caused by *Puccinia coronata* f. sp. A*venae* (*Pca*) is key for sustainable disease control. This study examined two recombinant inbred line populations, Provena x GS7 and Boyer x GS7, to identify adult plant resistance QTL in Australian fields. Seven distinct QTL associated with rust resistance were identified. KASP markers were developed for single nucleotide polymorphisms (SNPs) tightly linked to the four most significant QTL on chromosomes 4A and 7A. A major QTL named *QPc_GS7_4A.2* with a resistance allele derived from line GS7 was mapped to chromosome 4A, overlapping with genomic regions previously associated with both resistance gene *Pc61* and adult plant resistance. Genetic mapping for rust resistance at the seedling stage using a subset of Provena x GS7 lines with contrasting alleles at *QPc_GS7_4A.2* suggests a role of this locus in seedling resistance, which may be explained by the presence of *Pc61*. Seedling resistance profiles between GS7 and the Pc61 differential line against 20 *Pca* isolates, a haplotype analysis of *QPc_GS7_4A.2* in the oat crown rust differential set, and a collection of 182 oat lines support this hypothesis, although confirmation needs future research. The KASP markers developed in this study will assist breeders in efficiently integrating the resistance allele for gene combinations in new cultivars.

**Supplementary Information:**

The online version contains supplementary material available at 10.1007/s00122-025-05145-x.

## Introduction

Crown rust, a fungal disease caused by *Puccinia coronata* f. sp. *avenae* (*Pca*), is a significant threat to oat production worldwide (Nazareno et al. 2018; Simons 1985). Major resistance (*R*) genes conferring race-specific resistance against *Pca* have been widely used in oat breeding to safeguard crops from this disease. Such *R* genes usually encode specific receptors in the host that recognize effector molecules, which can vary between pathogen races, explaining the race-specificity (Dodds et al. 2024; Jones et al. 2024). This type of rust resistance is usually expressed from the seedling stage and lasts throughout the plant life cycle, so it is called all-stage resistance (ASR) (Periyannan et al. 2017). However, the effectiveness of ASR genes often diminishes over time due to the emergence of variants in the pathogen population that overcome resistance (Figueroa et al. 2020). Oat cultivars with ASR genes for crown rust typically lose resistance within five years of release, a pattern likely to persist with continued reliance on race-specific resistance genes (Carson 2008). This is particularly significant for cultivars that contain a single ASR gene, as the resistance can be overcome in as little as one cropping season. To combat this challenge, researchers and breeders have switched their focus to identifying and harnessing sources of more durable resistance and come up with strategies to stack multiple resistance genes/QTL.

Another class of rust *R* genes defined in cereals confer non-race-specific resistance associated with a partial resistance phenotype (Ellis et al. 2014; Periyannan et al. 2017). Partial resistance is considered highly effective in managing the disease because it slows down the evolution of pathogen virulence. Unlike race-specific ASR, partial resistance is not limited to specific pathogen races. While partial resistance manifests at the adult plant stages (therefore often referred to as adult plant resistance (APR), it is also possible to observe partial resistance at the seedling stage (Niks et al. 2015). The quantitative nature of partial resistance is explained by its mechanisms of action, which do not entirely inhibit fungal sporulation but can reduce pustule size, spore production, and prolong the latency period (Portyanko et al. 2005). Nevertheless, incorporating APR into breeding programs presents challenges due to its quantitative nature, and multiple loci must be combined to achieve high levels of resistance (Nazareno et al. 2022). Therefore, introgression of these novel alleles into elite germplasm is a lengthy process. In wheat, several major loci governing APR have been identified, offering potential shortcuts in breeding for durable disease resistance. For instance, the genes *Lr34* and *Lr67* encode membrane transporters that suppress rust growth independently of specific recognition (Krattinger et al. 2009, 2016; Milne et al. 2019; Moore et al. 2015).

In oat, of approximately 100 loci conferring resistance to *Pca* that have been cataloged to date, six are associated with APR (*Pc27*, *Pc28*, *Pc69*, *Pc72*, *Pc73*, *Pc74*). However, the chromosomal locations for all these genes are unknown (Carson 2017; Harder et al. 1984). Additional sources of APR have been postulated in various cultivars (Cabral et al. 2011; Heagle and Moore 1970; Luke et al. 1972; Upadhyaya and Baker 1962; Welsh et al. 1953). Although most of these APR sources remain uncharacterised, and their underlying genetic mechanisms are unknown. So far, few molecular markers associated with APR in oat have been developed for use in breeding and selection (Lin et al. 2014; Nazareno et al. 2022; Rines et al. 2018).

Previously, Babiker et al. (2015) identified three APR QTL in three mapping populations of recombinant inbred lines (RILs) derived from crossing the partially resistant sources, CDC Boyer (referred to as Boyer hereafter) and a breeding line 94197A1-9–2-2–2-5 (referred to as GS7 hereafter) with the susceptible cultivar Provena, and with each other (Boyer × GS7). In their study, Babiker et al. (2015) found that Boyer contributed the resistance alleles of two QTL located on chromosomes 12D (intervals of approximately 15.8 cM) and 19A (9.7 cM) (based on the cytology-based nomenclature by Sanz et al. 2010), while GS7 contributed one QTL on chromosome 13A (15.4 cM). These correspond to positions on chromosomes 2D, 4A, and 7A, respectively, under the uniform nomenclature system of Jellen et al. (2024).

This study aimed to evaluate the effectiveness of APR resistance loci from Boyer and GS7 under Australian field conditions using the same RIL populations. DArTSeq genotyping was employed to enhance marker density to resolve QTL regions. A total of seven distinct QTL associated with oat crown rust resistance were detected, and KASP markers were developed for single nucleotide polymorphisms (SNPs) tightly linked to the four most significant QTL on chromosomes 4A and 7A. KASP assays were implemented to assess the presence of the resistance alleles in an oat collection of 182 lines, including 150 lines from Nguyen et al. (2025b) and 32 lines postulated to carry APR. A strong QTL from GS7 linked to crown rust resistance was identified on homoeologous regions of chromosomes 4A and 4D, overlapping with genomic regions previously linked to the all-stage resistance gene *Pc61* and adult plant resistance. Genetic mapping for rust resistance at the seedling stage using a subset of Provena x GS7 RILs with contrasting alleles at *QPc_GS7_4A.2* supports a hypothesis that this locus contains a seedling resistance gene. Haplotype analysis using SNPs linked to this QTL in the differential set (Henningsen et al. 2024) and designed KASP markers in the oat collection identified the *QPc_GS7_4A.2* resistance haplotype as highly specific to *Pc61* carriers. The results suggest a strong linkage or potential identity between the *QPc_GS7_4A.2* on chromosome 4A and the *Pc61* locus, although further investigation is required. Overall, these findings underscore the potential of GS7 and Boyer as valuable sources of crown rust resistance, with the identified QTL and associated KASP markers providing insights to uncover underlying mechanisms and support marker-assisted selection in breeding programs.

## Materials and methods

### Plant material

The recombinant inbred lines (RILs-F_8_ generation) from the two mapping populations, ‘Provena x 94197A1-9-2-2-2-5’ and ‘CDC Boyer x 94197A1-9-2-2-2-5’, referred to as Provena x GS7 (*n* = 91) and Boyer x GS7 (*n* = 98), respectively, were developed and described by Babiker et al. (2015) through eight generations of selfing after the F_2_ generation. These RILs were sourced from the USDA Agricultural Research Service, Aberdeen, ID, USA. This study also includes an oat collection of 182 lines, comprising 32 lines postulated to carry APR and 150 lines recently compiled by Nguyen et al. (2024). These lines were sourced from USDA-ARS (St. Paul, MN, USA), the Australian Grain Genebank (AGG), and the CSIRO Avena seed stock. The list of plant materials is included in Supplementary File 1 Table S1 and Table S2.

### Disease resistance phenotyping

Field infection data for Provena x GS7 and Boyer x GS7 F_8_ RILs was collected from field trials in Australia at Manjimup, WA (33.24° S, 116.16° E), in 2023 and 2024, and Cobbitty, NSW (33.99° S, 150.69° E), in 2024. Data for the oat collection were recorded in Manjimup (2023) and Cobbitty (2024). All trials used six-row plots, Cobbitty (~ 20 cm rows) and Manjimup (~ 40 cm rows), with Row 1 as the spreader (oat lines Carrolup or Carrolup + Swan, both susceptible to *Pca*) and row 2–6 as entries. These trials were partially replicated (~ 1.2 reps per genotype) and included 10–15 replicated local checks with known resistance ratings at each year/site combination. In the Manjimup trials in 2023 and 2024 (MJ23 and MJ24), rust races (triplet codes as described by Park 2000) 0001–2 and 0005–0 were used, while in the Cobbitty trial 2024 (CB24), the pathotypes “4473–4,6,10, Bett, Barc”, “0767–3,4,5,6,10,Wa,Vo”, and “3707–1,4,5,6,7,10,12,Wa,Nu,Gw,Ge,Dr,Al” were utilized as they are highly frequent in the regions (Supplementary File 1 Table S3). Around the flowering between Zadoks growth stage GS59 and GS80 (Zadoks et al. 1974), rust infection severity was scored using the 0-to-100 modified Cobb scale (Peterson et al. 1948) in Manjimup, whereas in Cobbitty, severity was rated on a 1–9 scale. To enable comparison between the two locations, the 1–9 scale scores from CB24 were converted into percent severities following the method of Bariana et al. (2007).

Rust infection assays at the seedling stage were conducted in growth cabinets on a subset of 30 RILs from the Provena x GS7 population, which were selected for having contrasting alleles at the QTL *QPc_GS7_4A.2* identified through field-based QTL mapping. These lines were tested against *Pca* isolate 22WA54 (Supplementary File 1 Table S4), which was virulent Provena but avirulent to GS7 to evaluate the effect of the locus *QPc_GS7_4A.2* at the seedling stage. In addition, similar seedling assays were also performed on GS7, Provena, Pc60, Pc61, and Swan oat lines against 20 *Pca* isolates (Supplementary File 1 Table S5) to compare their resistance profiles (Henningsen et al. 2024; Nguyen et al. 2025b). Plants were grown under controlled conditions (23/18 °C, 16/8 h, light/dark), and *Pca* spore samples were applied to plants 10 days after sowing, and infection scores were recorded 10 days post-inoculation. Seedling infection and scoring methods were described by Miller et al. (2020), with infection type scores (“0”, “0;”, “;”, “;C”, “1;”, “1”, “2”, “3”, “3 + ”, “4”) converted to a 0–9 numeric scale (0, 1, 2, 3, 4, 5, 6, 7, 8, 9) respectively for plotting as heatmaps using ComplexHeatmap v2.20.0 in R v4.4.0 (Gu 2022).

Broad-sense heritability (H^2^) for rust severity was estimated for each RIL population within each environment using linear mixed models fitted by restricted maximum likelihood (REML) in R (lme4 package), with genotypes as random effects and checks as fixed. Genotypic (σ^2^g) and residual (σ^2^e) variances were extracted to calculate heritability (H^2^ = σ^2^g / (σ^2^g + σ^2^e / r), where *r* is the number of replicates (= 1 in this augmented design). For multi-environment analysis (MJ23, MJ24, CB24), a mixed model (*lmer(Rust Severity* ~ *Environment* + *(1|RILs)*)) was used, treating RILs as random and environment as fixed. Best linear unbiased predictions (BLUPs) were extracted as adjusted phenotypes for QTL mapping, and across-environment H^2^ was computed as H^2^ = *σ*^2^g / (*σ*^2^g + *σ*^2^e / n), where *n* is the number of environments.

### Genotyping

Five seeds from each oat line were sent to Diversity Arrays Technology Pty Ltd. (Canberra, Australia; https://www.diversityarrays.com/) for DNA extraction and genotyping using their proprietary genome complexity reduction-based sequencing technology. To identify SNP markers, the sequences obtained from DArTSeq were aligned against the reference genome sequence *Avena sativa* OT3098 v2 (PepsiCo, https://wheat.pw.usda.gov/jb?data=/ggds/oat-ot3098v2-pepsico), deposited in the GrainGenes database (Yao et al. 2022), using BLAST (Altschul et al. 1990) with an expected value (E) threshold of less than 5e-7 and sequence identity greater than 70%. Only markers that matched a genomic location were kept. The SNP dataset was filtered in dartR (v2.9.7) by first removing loci with all missing data, then excluding monomorphic loci to retain only polymorphic markers (Gruber et al. 2018). A call rate filter removed loci with over 50% missing data, and markers with a minor allele frequency below 0.01 were discarded. The genotypic data of Provena x GS7 and Boyer x GS7 RILs were transformed to a parent-based format (ABH) by using the GenosToABH plugin from TASSEL (v.5.2.64), using the codes A: male parent, B: female parent, H: heterozygous. Data imputation was conducted using ABHgenotypeR v.1.0.1 R package (Furuta et al. 2017).

### Linkage map construction for Provena x GS7 and Boyer x GS7 RILs

Linkage group construction was carried out using the “mstmap” algorithm implemented in the R package ASMap v.1.0.7 R package (Taylor and Butler 2017). The initial group assignment was based on *p*-value thresholds of 1e-19 for Provena x GS7 and 1e-21 for Boyer x GS7, with the “Kosambi” function used for genetic distances. Markers not assigned to linkage groups were removed. Subsequently, chromosome names were assigned to linkage groups by identifying the most frequent chromosome location of DArTSeq markers mapped to the OT3098 v2 reference genome. Linkage groups with the same chromosome name were then merged using the “mergeCross” function with a gap threshold of 10 cM, and marker order was refined through a second round of “mstmap” using a p-value threshold of 1e-6. Linkage groups with zero length or fewer than seven markers were filtered out. Unmerged linkages were merged with other already-merged linkage groups sharing the same chromosome name using a permissive p-value threshold of 1e-2, considering orientation and correlation with the physical map. Genetic distances were recalculated using the “Kosambi” function after merging. The correlation between genetic map marker order and the reference genome was calculated using a Pearson’s correlation test and visualized with the “ggplot2” R package (Wickham 2016).

### QTL mapping

QTL mapping was performed for the Provena x GS7 and Boyer x GS7 RILs using the genetic maps in combination with field phenotypic data from individual environments and BLUPs estimated across environments. For the subset of RILs from Provena x GS7 (*n* = 30), mapping was carried out using the genetic map and seedling resistance data. All analyses were conducted using composite interval mapping (CIM) with the R/qtl package (v1.66) (Haley–Knott with forward selection to three markers and a window size of 10 cM) (Broman and Sen 2009). The threshold for the logarithm of odds (LOD) for significant QTL declaration was defined by 1000 permutations at *p* ≤ 0.05. The custom interactive R script for QTL mapping, developed in RStudio 2024.04.2, is based on the R/qtl manual by Broman and Sen (2009). The proportion of phenotypic variance explained (% var) was obtained by fitting multiple-QTL models using the *fitqtl* function in the R/QTL package. Putative QTL identified from initial scans were fitted simultaneously, with the total model % var representing variance jointly explained by all QTL and partial % var values obtained by dropping each locus in turn to assess its contribution (Broman et al. 2003).

### Comparative mapping analysis

The flanking sequences of significant markers associated with QTL from previous studies (Babiker et al. 2015; Klos et al. 2017; Nazareno et al. 2022; Rines et al. 2018; Wight et al. 2004) were searched against the reference genome OT3098 v2 by BLAST using Geneious Prime® 2022.2.2. The flanking sequences of markers were taken from NCBI GenBank (https://www.ncbi.nlm.nih.gov/nuccore/) and T3/Oat (https://oat.triticeaetoolbox.org/). Syntenic relationships between chr4A and 4D were established and visualized through a Comparative Genomics Platform (CoGe; https://genomevolution.org/coge/SynMap.pl) and the SynMap2 tool (Haug-Baltzell et al. 2017).

### KASP marker development

We designed KASP markers for the SNPs closely linked to identified QTL: *SNP-350765064_4A*, *SNP-351486359_4A*, and *SNP-352361337_4A* for *QPc_GS7_4A.1, SNP-459779133_4A, SNP-459972095_4A,* and *SNP-460562184_4A* for *QPc_GS7_4A.2, SNP-4039736_7A* and *SNP-411798_7A* for *QPc_GS7_7A,* and *SNP-67318388_7A* and *SNP-69787185_7A* for *QPc_Provena_7A/QPc_Boyer_7A*. The flanking sequences of these SNPs were imported into the Kraken™ software system for marker design using the default parameters (LGC Biosearch Technologies, UK; https://www.biosearchtech.com/). KASP assays were performed in the SNPline PCR Genotyping System (LGC, Middlesex, United Kingdom), following the methods described by Shi et al. (2023). KASP primer sequences are included in Supplementary File 1 Table S6 To assess KASP marker predictive ability, we used the *confusionMatrix* function from the R package “caret” (https://github.com/topepo/caret/) to compare marker-based genotype predictions with observed phenotypic responses, reporting sensitivity, specificity, and accuracy. The crown rust infection severity (CRS) was converted into binary classes: resistant (CRS < 50%) and susceptible (CRS ≥ 50%). Marker alleles were assigned to resistant or susceptible classes according to prior mapping results.

### Haplotype visualization

Haplotype of QTL *QPc_GS7_4A.2* identified in chr4A from mapping analysis was visualized in the oat collection and the differential set using Flapjack (Milne et al. 2010). The *QPc_GS7_4A.2* haplotype in the oat collection is visualized based on the result of KASP genotyping assays of *KASP_4A_459779133,* KASP_4A_459972095, and KASP_4A_460562184; and the *QPc_GS7_4A.2* haplotype in the differential set (Henningsen et al. 2024) was observed based on the genotype of SNP markers *SNP-459779133_4A, SNP-459972095_4A*, and *SNP-460562184_4A* taken from DArTSeq genotypic data (Nguyen et al. 2024).

### Pedigree evaluations

The pedigree records of oat lines of interest were obtained from Fitzsimmons et al. (1983), and the T3/Oat database (Morales et al. 2022), with information extracted from the “Pedigrees of Oat Lines” POOL database (https://triticeaetoolbox.org/POOL/index_db.php; Tinker and Deyl 2005).

## Results

### Field assessment of rust infection severity

The *Pca* race used in Manjimup fields 2023 and 2024 (MJ23 and MJ24) was 0001-2 and 0005-0, respectively, while the Cobbitty 2024 (CB24) nursery included a mix of races: 0767-3,4,5,6,10,Wa, Vo; 3707-1,4,5,6,7,10,12,Wa,Nu,Gw,Ge,Dr,Al; and 4473-4,6,10, Bett, Barc. The virulence profiles of these races were determined by nursery managers, following the methods described by Park (2000) and presented in Supplementary File 1 Table S3. The rust races in CB24 were broadly virulent, exhibiting virulence against 29 differential lines, including Pc61. In contrast, the races in MJ23 and MJ24 are virulent to only Swan, Pc46, and Pc38 in MJ23, and Swan and Pc71 in MJ24.

**Fig. 1:**
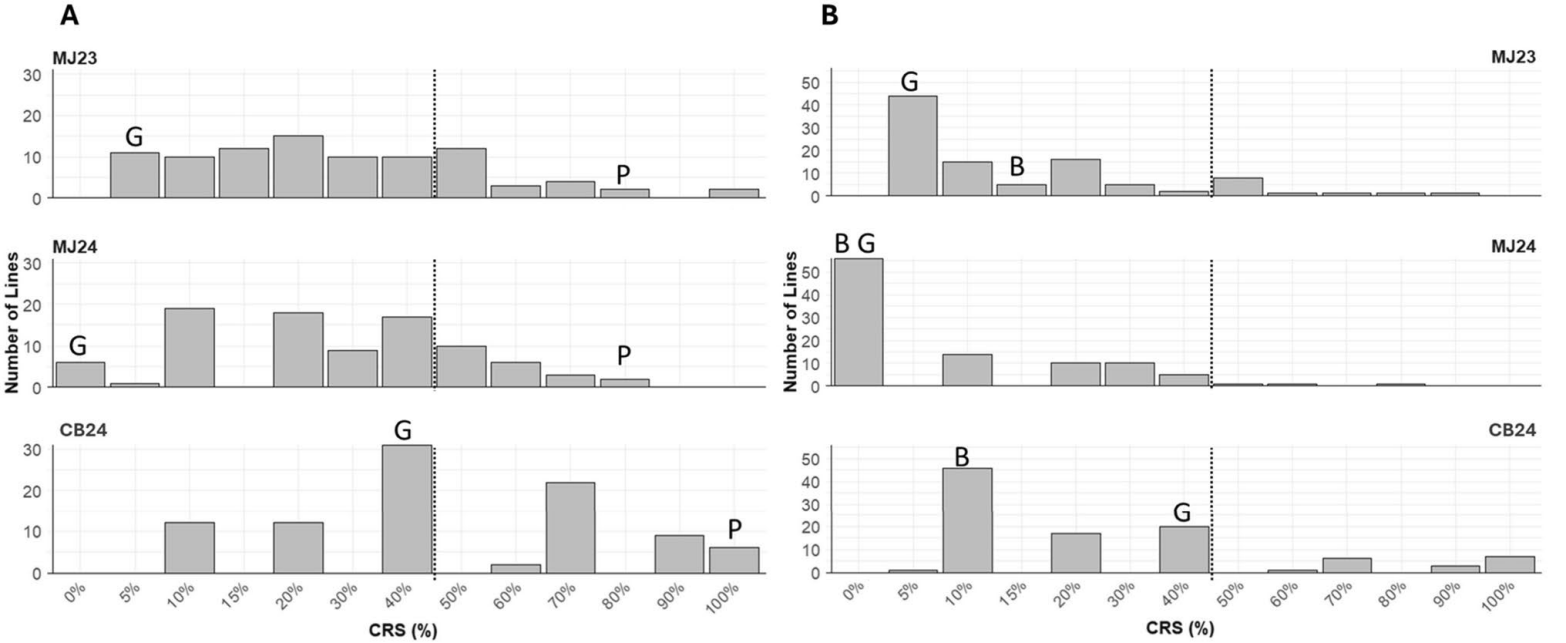
Histogram of field infection severity data from Manjimup 2023 (MJ23), 2024 (MJ24), and Cobbitty 2024 (CB24) of (**A**) Provena (P) x GS7 (G) RILs (*n* = 91); (**B**) Boyer (B) x GS7 (G) RILs (*n* = 98). The x-axis represents crown rust severity = CRS, CRS < 50% = resistance and CRS ≥ 50% = susceptibility, whereas the y-axis represents the number of lines. The dashed lines separate resistance (left) and susceptibility (right)

The RILs from Provena x GS7 and Boyer x GS7 populations (F_8_ generation) and the parental lines were scored for crown rust infection severity (CRS) at the flowering stage in MJ23 and MJ24, and CB24. The data showed rust resistance trait segregation (Fig. 1), with a high correlation between crown rust severity percentage in MJ23 and MJ24 for both populations, Provena x GS7 (r = 0.72) and Boyer x GS7 (0.58) (Supplementary File 1 Table S7). In contrast, lower correlations were observed between the Manjimup trials (MJ23 and MJ24) and the Cobbitty trial (CB24) (r ≤ 3.5). Rust severity was higher in Cobbitty than in Manjimup, as a greater number of susceptible lines were observed in Cobbitty (Fig. 1). Among the parental lines, Provena remained consistently susceptible across trials (CRS = 80–100%), Boyer showed high resistance in all trials (CRS = 0–15%), and GS7 exhibited strong resistance in MJ23 and MJ24 trials (CRS = 0–5%) but displayed a moderate resistance-to-moderate susceptibility in CB24 (CRS = 40%). Across all trials, a greater number of lines with low crown rust severity (CRS = 0 – 40%) were observed in the Boyer x GS7 population, compared to Provena x GS7 (Fig. 1), likely reflecting the previously reported combined resistance contribution from both parental lines, Boyer and GS7 (Babiker et al. 2015).

Broad-sense heritability (H^2^) for rust severity ranged from moderate to high (0.38–0.95) across the two RIL populations (Supplementary File 1 Table S8), with Provena × GS7 consistently showing higher values than Boyer × GS7. Heritability was highest at CB24 (0.95 for both) and lowest at MJ24 (0.38–0.69). From the multi-environment analysis, genotypic effects explained 27% and 30% of phenotypic variance in Boyer × GS7 and Provena × GS7, respectively, corresponding to moderate heritability (H^2^ = 0.52–0.56). These findings suggest that, in addition to genotypic effects, a substantial proportion of the variation in rust resistance scores in both populations is driven by differences among rust isolates/mixtures across environments.

### Construction of genetic maps of Provena x GS7 and Boyer x GS7 RILs

**Fig. 2:**
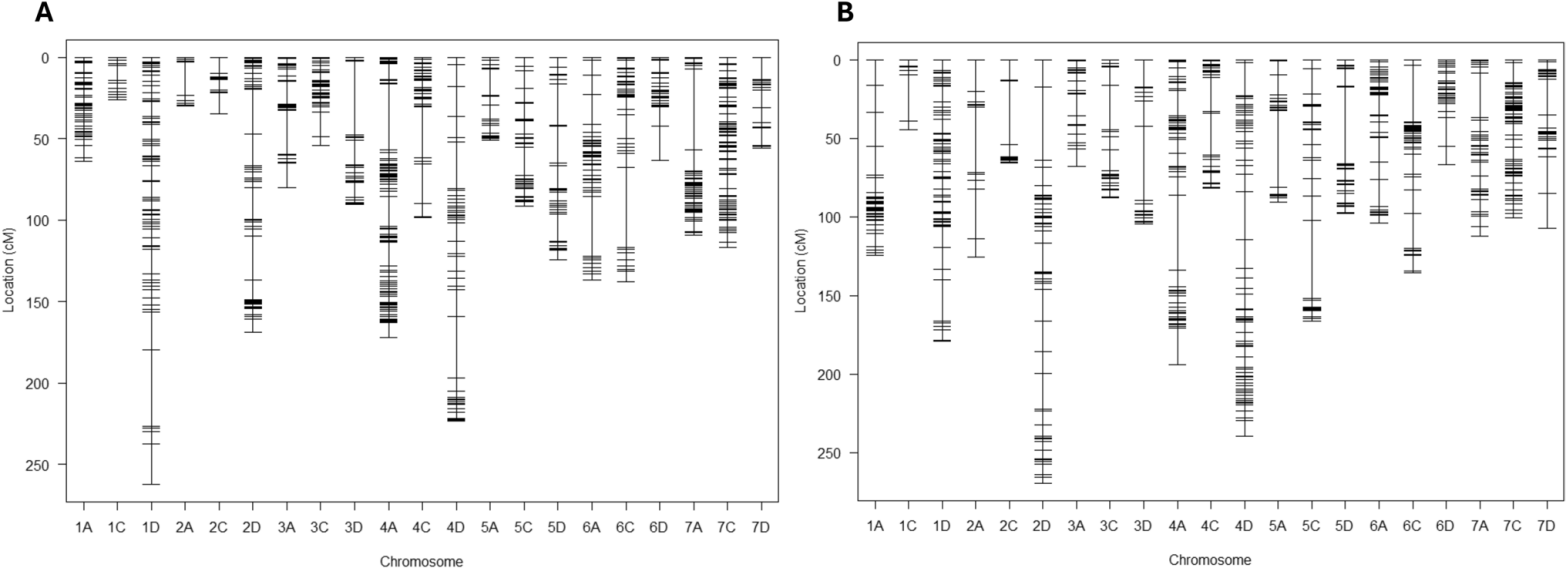
(**A**) Distribution of markers (4493 SNPs) across 21 oat chromosomes in the genetic map of the Provena × GS7 RIL population (*n* = 91); (**B**) Distribution of markers (5048 SNPs) across 21 oat chromosomes in the genetic map of the Boyer × GS7 RIL population (*n* = 98). The x-axis represents the 21 oat chromosomes anchored to the genetic maps, while the y-axis shows the genetic positions of markers in centimorgans (cM)

The RILs from Provena x GS7 and Boyer x GS7 mapping populations and the parental lines were genotyped using DArTSeq, generating 31,101 and 26,385 segregating SNPs, respectively. After filtering and converting to a parent-based format (ABH), 5,931 and 6,386 markers were used to construct a genetic map for each family. The final genetic maps included 4,493 and 5,048 SNPs for Provena x GS7 and Boyer x GS7, respectively, with 2,323 common markers between the two populations (Fig. 2 and Supplementary File 2). The genetic maps spanned 2191.5 cM and 2568.2 cM, encompassing 43 and 40 linkage groups with an average marker spacing of 0.5 cM in both maps (Supplementary File 2 and Supplementary File 3 Fig. S1). The DArTSeq markers were also assigned to genomic locations in the *Avena sativa* OT3098 v2 reference genome sequence based on sequence alignment. A high proportion of markers within the same linkage group aligned to the same chromosome on the physical map (OT3098 v2): 96.75% for Provena x GS7 (Supplementary File 3 Fig. S2) and 95% for Boyer x GS7 (Supplementary File 3 Fig. S3). The average correlation coefficient between genetic and physical map orders for 21 chromosomes was r = 0.86 in both populations. This allowed the genetic maps to be anchored to the 21 oat chromosomes using ASMap R package (Taylor and Butler 2017), and each map provided close to complete coverage of the genome (Fig. 2). Chr1D showed the longest linkage group in Provena x GS7 (262.3 cM), while chr2D was the longest in Boyer x GS7 (269.8 cM) (Fig. 2).

### Identification of QTL associated with oat crown rust severity

#### Provena x GS7 population

**Fig. 3:**
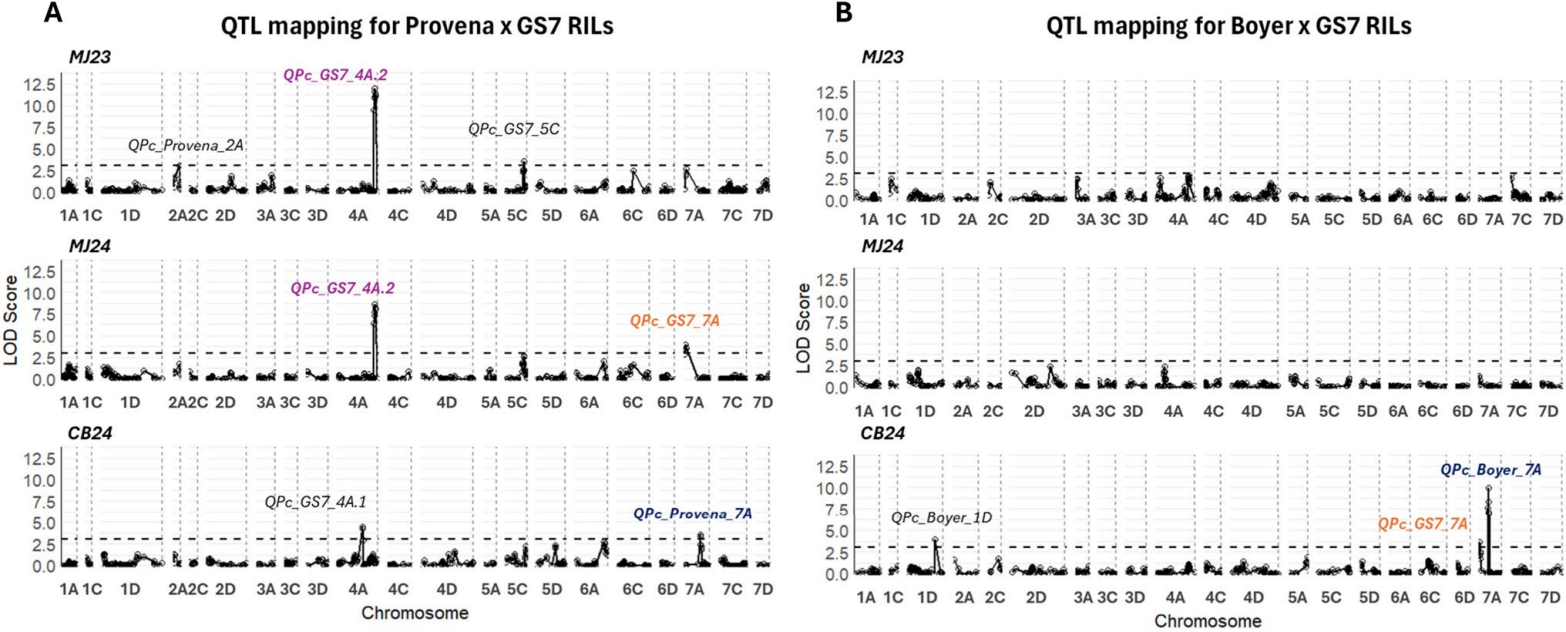
QTL mapping in RIL populations according to field trial. Plots for Provena x GS7 (**A**) and Boyer x GS7 (**B**) mapping populations at Manjimup in 2023 (MJ23), 2024 (MJ24), and Cobbitty 2024 (CB24) are shown with chromosome numbers in the x-axis and LOD score in the y-axis. LOD threshold (dash lines) = 3. The colors indicate co-located QTL

QTL analysis identified significant peaks on chr2A (*QPc_Provena_2A*), 4A (*QPc_GS7_4A.1* and *QPc_GS7_4A.2*), 5C (*QPc_GS7_5C*), and 7A (*QPc_GS7_7A* and *QPc_Provena_7A*), with the majority of them carrying resistance alleles from GS7. The exceptions were *QPc_Provena_2A* and *QPc_Provena_7A*, which have the resistance allele from Provena (Fig. 3A and Table 1). *QPc_GS7_4A.2*, located between 158.00 and 162.16 cM, was the only QTL detected in two trials (MJ23 and MJ24) but showed the strongest significance (Fig. 3A), accounting for 26.36% of the phenotypic variance explained (% var) in MJ23 and 26.69% in MJ24 (Table 1). The second most significant detected QTL, *QPc_GS7_4A.1* (LOD = 4.38; % var = 17.56), which appeared only in the CB24 trial, was located on chr4A between 85.36 and 104.09 cM, approximately 54 cM away from *QPc_GS7_4A.2*. Low linkage disequilibrium (LD) was found between *QPc_GS7_4A.1* and *QPc_GS7_4A.2* (Supplementary File 3 Fig. S4A). The two minor QTL on chr7A, *QPc_GS7_7A* and *QPc_Provena_7A*, were identified in different trials and have low LD to each other (Supplementary File 3 Fig. S4B). The *QPc_GS7_7A*, carrying a favorable allele from GS7, was detected in MJ24, whereas *QPc_Provena_7A*, derived from the susceptible parent Provena, was identified in CB24. The two other QTL, *QPc_Provena_2A* and *QPc_GS7_5C*, were both significant in MJ23. *QPc_Provena_2A* was located between 2.89 and 26.4 cM on chr2A, while QPc_GS7_5C was located on chr5C, with % var values of 5.55% and 6.34%, respectively. Two loci, *QPc_GS7_4A.2* and *QPc_GS7_5C*, were also identified from QTL analysis using BLUPs derived from the multi-environment analysis (Supplementary File 3 Fig. S5, Table 1, Supplementary File 4). This is consistent with those previously detected using phenotypic data from single environments. Additionally, a novel QTL, *QPc_Provena_6A* (at 85.4–129.4 cM, with % var values of 10.50%) (Supplementary File 3 Fig. S5, Table 1), was detected using BLUP values. However, it was not significant in earlier analyses (Fig. 3A), indicating that multi-environment integration enhanced the statistical power of detection.

**Table 1:** Summary of composite interval mapping in Provena x GS7 and Boyer x GS7 RILs for crown rust severity in Manjmup 2023 (MJ23)—2024 (MJ24), Cobbitty 2024 (CB24), and multiple environment analysis (MET). Number of * indicates co-located QTL. % var: proportion of phenotypic variance explained

Population	QTL	Chromosome	QTL region			LOD	% var	Field Trial
			Peak Marker	Peak (cM)	Interval (cM)			
Provena x GS7	*QPc_Provena_2A*	2A	SNP-313607169_2A	23.46	2.89—26.4	3.06	5.55	MJ23
	*QPc_GS7_4A.1*	4A	SNP-352361337_4A	104.09	85.36—104.09	4.38	17.56	Cob24
	QPc_GS7_4A.2*	4A	SNP-459972095_4A	161.6	158.00—162.16	11.95–8.18	26.36 – 26.69—28.22	MJ23—MJ24—MET
	*QPc_GS7_5C*	5C	SNP-536888142_5C	80.76	75.35 -85.76	3.58	6.34—10.71	MJ23—MET
	*QPc_Provena_6A*	6A	SNP-407338366_6A	122.03	85.4—129.4	3.91	13.4	MET
	QPc_GS7_7A**	7A	SNP-4039736_7A	3.6	0.00—7.03	4.01	16.71	MJ24
	QPc_Provena_7A***	7A	SNP-66719542_7A	70.44	69.88—74.38	3.56	17.34	Cob24
Boyer x GS7	*QPc_Boyer_1D*	1D	SNP-432451680_1D	140.02	133.41—166.12	3.89	11.12	Cob24
	QPc_GS7_7A**	7A	SNP-4039736_7A	0.64	0.00—1.92	3.57	8.95	Cob24
	QPc_Boyer_7A***	7A	SNP-66719542_7A	49.1	48.3—50.14	9.98	30.17—13.40	Cob24—MET

#### Boyer x GS7 population

Three significant QTL were identified in the Boyer x GS7 RILs on ch1D (*QPc_Boyer_1D* with % var = 11.12%) and 7A (*QPc_GS7_7A* with % var = 8.95% and *QPc_Boyer_7A* with % var = 30.17%) (Fig. 3B). All were detected in CB24, while none were identified in MJ23 or MJ24, likely due to the high resistance of most lines, with only a few RILs exhibiting susceptibility in Manjimup trials (Fig. 1). The *QPc_GS7_7A* identified in this population, located at 0.00–1.92 cM and carrying a favorable allele from GS7, corresponds to the same QTL identified in the Provena x GS7 population (Table 1). Similarly, *QPc_Boyer_7A* mapped between 48.3 and 50.14 cM, with a resistance allele from Boyer, co-located with *QPc_Provena_7A*, previously identified in the Provena x GS7 population. Using BLUPs from across environments in QTL analysis, the locus *QPc_Boyer_7A* was also detected in this population, with % var value of 13.40% (Supplementary File 3 Fig. S5, Table 1, Supplementary File 4).

### KASP marker development and screening of the oat collection

**Fig. 4:**
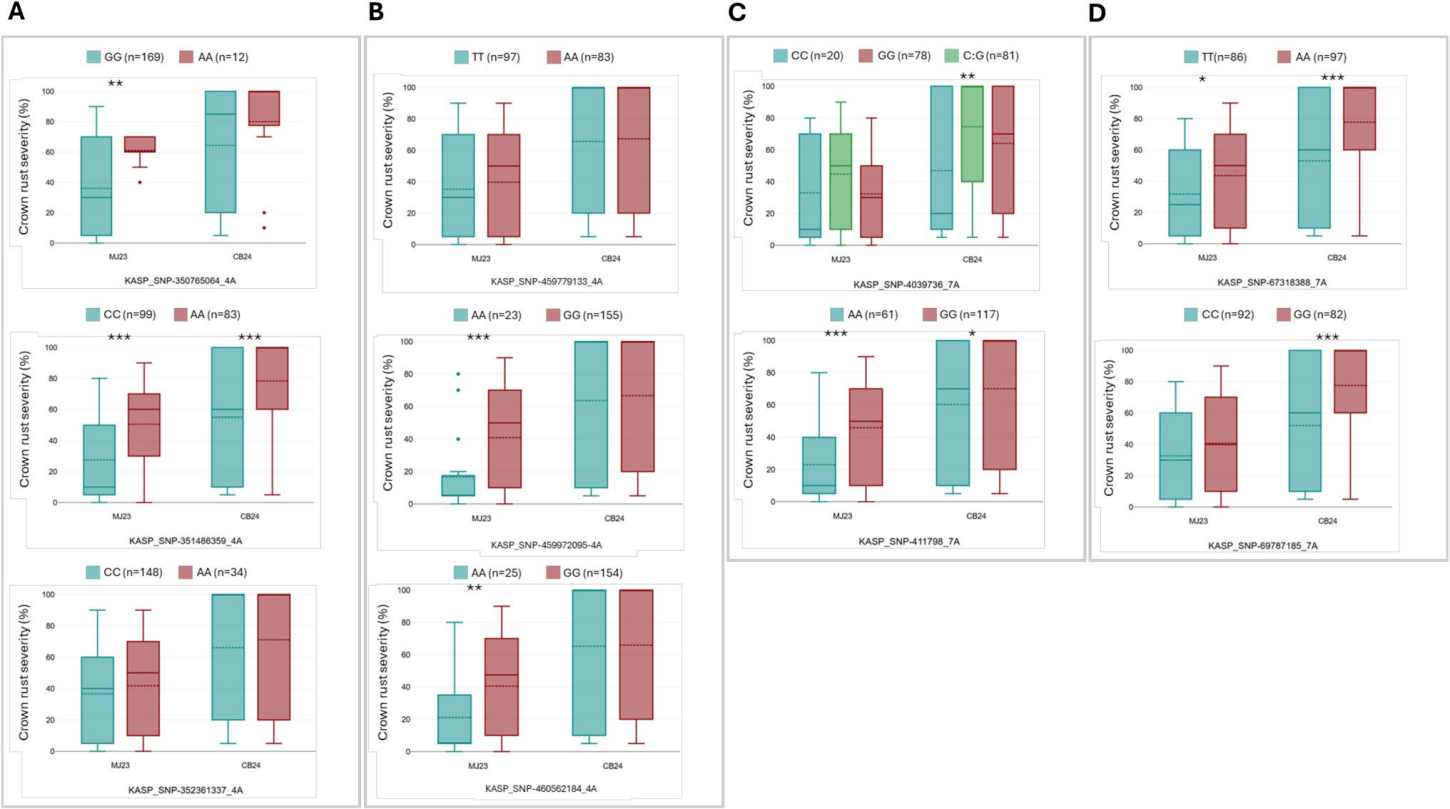
Phenotypic effect of KASPs associated with QTL A QPc_GS7_4A.1; B QPc_GS7_4A.2; C QPc_GS7_7A; D QPc_Provena_7A/QPc_Boyer_7A in the oat collection (*n* = 182). Pairwise t-tests were used to compare the allelic effect on rust severity scores in Manjimup 2023 (MJ23) and Cobbitty 2024 (CB24); significant levels are based on *p < 0.05, **p < 0.01, ***p < 0.001. The teal color indicates the resistant alleles, the rose indicates the susceptible alleles, and the green indicates the heterozygous allele. The boxes’ solid and dash lines indicate median and mean, respectively (color figure online)

Several SNPs that were significantly associated with *QPc_GS7_4A.1*, *QPc_GS7_4A.2*, *QPc_GS7_7A*, and *QPc_Provena_7A*/*QPc_Boyer_7A* were successfully converted into KASP markers, including *KASP_SNP-350765064_4A, KASP_SNP-351486359_4A,* and *KASP_SNP-352361337_4A* for *QPc_GS7_4A.1*, *KASP_SNP-459779133_4A*, *KASP_SNP-459972095_4A*, and *KASP_SNP-460562184_4A* for *QPc_GS7_4A.2*, *KASP_SNP-4039736_7A* and *KASP_SNP-411798_7A* for *QPc_GS7_7A*, and *KASP_SNP-67318388_7A* and *KASP_SNP-69787185_7A* for *QPc_Provena_7A*/*QPc_Boyer_7A* (Supplementary File 1 Table S6). KASP marker assays were performed to assess the presence of these QTL in an oat collection of 182 oat lines (Supplementary File 1 Table S9). The results were integrated with rust severity scores of the oat collection from MJ23 and CB24, and a *t*-test was conducted to evaluate allelic effects on the phenotype (Fig. 4). The markers *KASP_SNP-351486359_4A* (*QPc_GS7_4A.1*), *KASP_SNP-411798_7A* (*QPc_GS7_7A*), and *KASP_SNP-67318388_7A* (*QPc_Provena_7A*/*QPc_Boyer_7A*) showed consistent associations with crown rust severity in the oat collection across both MJ23 and CB24 trials (Fig. 4). In contrast, the markers for *QPc_GS7_4A.2*, *KASP_SNP-459972095_4A* and *KASP_SNP-460562184_4A* were only associated with resistance scores in MJ23 but not CB24, aligning with QTL mapping results that identified this QTL only in MJ23 and MJ24. The predictability of KASP markers KASP_SNP-351486359_4A, KASP_SNP-411798_7A, and KASP_SNP-67318388_7A across the two field environments (MJ23 and CB24) was further assessed through a confusion matrix analysis (Supplementary File 3 Fig. S6A). SNP_351486359_4A showed the highest sensitivity (0.69–0.75) and moderate accuracy (0.64–0.66), making it most reliable for detecting resistant lines (Supplementary File 3 Fig. S6B). SNP_411798_7A achieved the highest specificity (0.71–0.82) but lower sensitivity (0.43–0.51), while SNP_67318388_7A showed balanced but moderate accuracy (0.55–0.65), performing better in CB24 than MJ23.

#### QTL QPc_GS7_4A.2 is colocalized with genomic regions associated with adult plant resistance and other race-specific resistance

**Fig. 5:**
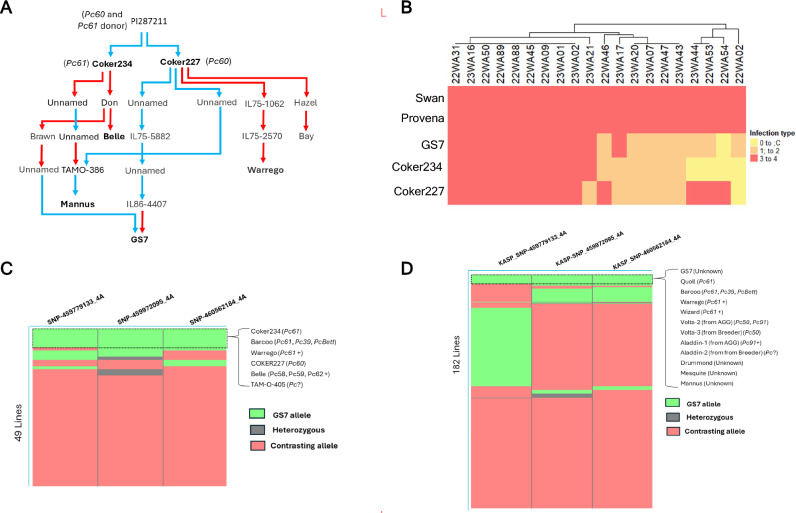
(**A**) Pedigree relationship of GS7 and Coker234 (Pc61) and Coker227 (Pc60). Pedigrees were obtained from “Pedigrees of Oat Lines” POOL database (https://triticeaetoolbox.org/POOL; Tinker and Deyl 2005) and Fitzsimmons et al. (1983). Red lines indicate a maternal relationship, and blue indicates a paternal relationship. Names of oat lines in bold represent lines that carry the resistance haplotype at QPc_GS7_4A.2. (**B**) Heatmap comparing virulence profiles of 20 Pca isolates on GS7, Provena, Pc61, and Pc60. Swan is a widely susceptible cultivar used as the susceptible control. The color range indicates the infection type of isolate on host: resistant (0 to 2) to susceptible (3 to 4). C Presence of haplotype 4A_GS7 in the oat differential set (*n* = 49) and D the oat collection (*n* = 182). The markers on top are the most significant SNP at the QTL region on chr4A taken from DArTSeq in the differential set, converted to KASP, and tested in the oat collection. Colour indicates the allele of GS7 (green), heterozygous (gray), and the contrasting allele (rose) (color figure online)

To further explore the most significant QTL *QPc_GS7_4A.2* derived from GS7 on chr4A, we examined its colocalization with known resistance loci through comparative mapping analysis. A BLAST search of the flanking sequences of markers significantly associated with rust resistance from previous studies (Babiker et al. 2015; Klos et al. 2017; Nazareno et al. 2022) identified significant matches (% identity > 90.1 and e-value < 2.55E-18) on both chr4A and 4D in reference genome OT3098 v2 (Supplementary File 1 Table S10 and Supplementary File 3 Fig. S7). Additionally, some markers physically mapped to chr4D in our QTL *QPc_GS7_4A.2* overlap with the syntenic APR locus *QPc.APR4D.2* identified by Nazareno et al. (2022) on chr4D (Supplementary File 3 Fig. S7A). Synteny analysis revealed significant homology across most regions of chr4A and 4D (Supplementary File 3 Fig. S7B). The *QPc_GS7_4A.2* was found to overlap with the genomic region associated with the seedling resistance gene *Pc61* and APR loci identified by Klos et al. (2017) and Nazareno et al. (2022), respectively (Supplementary File 3 Fig. S7A). Pedigree connections were found between GS7 and both Coker234 (*Pc61*) and Coker227 (*Pc60*) (Fig. 5A).

Given that *QPc_GS7_4A.2* overlapped with the known *Pc61* locus and manifested only in the Manjimup field across both the Provena x GS7 mapping population and the oat collection, along with GS7’s pedigree connection to both Coker234 (*Pc61*) and Coker227 (*Pc60*), we examined if this QTL may associate with a seedling resistance gene. A subset of 30 RILs from the Provena x GS7 population, carrying contrasting alleles at the *QPc_GS7_4A.2* locus (15 with the resistance allele and 15 with the susceptible allele), were selected for seedling resistance testing against the rust isolate 22WA54. The isolate 22WA54 is avirulent to *Pc61* and *Pc60* (Supplementary File 1 Table S4; Henningsen et al. 2024). The results showed that GS7 and the 15 RILs carrying the *QPc_GS7_4A.2* resistance allele exhibited resistance to 22WA54, whereas Provena and the 15 RILs carrying the *QPc_GS7_4A.2* susceptible allele were all susceptible (Supplementary File 1 Table S11). QTL analysis using the seedling resistance data from this subset of samples identified only a single peak that co-mapped with the *QPc_GS7_4A.2* locus (Supplementary File Fig. S8), confirming a role of *QPc_GS7_4A.2* as an ASR locus.

Another rust resistance phenotyping experiment at the seedling stage was conducted to compare the resistance profiles of Provena, GS7, Pc60, and Pc61. The experiment used 20 contemporary *Pca* isolates collected from WA in 2022 and 2023 (Supplementary File 1 Table S5**;** Henningsen et al. 2024; Nguyen et al. 2025b). The results indicated that Provena was susceptible to all tested isolates, while GS7 exhibited seedling resistance to nine *Pca* isolates, confirming it carries at least one ASR gene (Fig. 5B). Additionally, GS7 displayed a highly similar resistance profile to Coker234 (*Pc61* differential line), differing only for one isolate.

To assess the specificity of *QPc_GS7_4A.2* in the oat crown rust differential set (*n* = 49), previously used in *Pca* virulence surveillance (Henningsen et al. 2024; Nguyen et al. 2025b), we examined the presence of *QPc_GS7_4A.2* resistance haplotype in these lines (Fig. 5C). The resistance haplotype of *QPc_GS7_4A.2* is defined by three SNPs previously used to develop KASP assays for *QPc_GS7_4A.2*, *SNP-459779133_4A*, *SNP-459972095_4A*, and *SNP-460562184_4A* that were also found in the DArTSeq data of the oat crown rust differential set (Nguyen et al. 2024, 2025a). In the differential set, the resistance haplotype of *QPc_GS7_4A.2* was present in six lines, for which the presence of certain race-specific genes has been postulated based on pathogenicity assays (Fig. 5C). These lines include Coker234 (*Pc61* differential line), Coker227 (*Pc60* differential line), Warrego (*Pc61* +), Barcoo (*Pc61*, *Pc39*, *PcBett*), Belle (*Pc58*, *Pc59*, *Pc62*, *Pc?*), and TAM-O-405 (Unknown resistance) (Carson 2017; Forsberg et al. 1999; Park et al. 2009). Belle also has a pedigree connection to Coker24 (*Pc61*) (Fig. 5A).

In the oat collection, the resistance haplotype of *QPc_GS7_4A.2* was found in 12 lines through genotyping with KASP markers *KASP_SNP-459779133_4A*, *KASP_SNP-459972095_4A*, and *KASP_SNP-460562184_4A* (Fig. 5D and Supplementary File 1 Table S9). In addition to GS7, eleven other oat lines carried the *4A_GS7* resistance haplotype, including Quoll (*Pc61*), Warrego (*Pc61* +), Barcoo (*Pc61*, *Pc39*, *PcBett*), Wizard (*Pc61*) (Park et al. 2009; Park 2013; Cuddy et al. 2016), Volta-2 (*Pc50*, *Pc91*), Volta-3 (*Pc50*), Aladdin-1 (*Pc91*), Aladdin-2 (unknown *Pc* gene(s)) (Park 2013; Nguyen et al. 2025a), Mesquite, Drummond (*Pc39*) (Nguyen et al. 2024, 2025a), and Mannus. Among these, Mannus was found to have a pedigree connection with Coker234 (*Pc61*) (Fig. 5A). Notably, Mesquite is the only postulated APR line in the oat collection that was found to carry the resistance haplotype of *QPc_GS7_4A.2* (Supplementary File 1 Table S9). Previously, a QTL in Mesquite was mapped to chr4A by Nazareno et al. (2022), which is 50 Mb from *QPc_GS7_4A.2* (Supplementary File 3 Fig. S7A). Two other lines, Amarela and NMO 877, which also had APR QTL mapped to the same region of *QPc_GS7_4A.2* on chr4A (Nazareno et al. 2022) (Supplementary File 3 Fig. S7A), differed from the *QPc_GS7_4A.2* resistance haplotype by one marker (Supplementary File 1 Table S9).

The *QPc_GS7_4A.2* interval (~ 5 Mbp on chr4A, 456,300,687–461,488,017 bp) in the *A. sativa* OT3098 v2 genome contains 69 annotated genes, including 25 related to disease resistance (Supplementary File 1 Table S12). Notably, a cluster of 10 Disease Resistance Protein RGA5 genes (458,178,696–459,022,508 bp) and a tandem of three ACCELERATED CELL DEATH 6 (ACD6) genes were identified. Other potential candidates include Ankyrin repeat-containing protein NPR4, WRKY transcription factor 49, Receptor-like cytoplasmic kinase 176, and Disease resistance protein Pik-2**.**

## Discussion

This study employed two established RIL mapping F_8_ populations (Babiker et al. 2015) derived from APR carrying lines, GS7 and Boyer, and a susceptible cultivar (Provena) to assess the effectiveness of the postulated APR loci present in these families under Australian field conditions. Both Boyer and GS7 were confirmed to exhibit high levels of resistance to oat crown rust, while Provena was highly susceptible. QTL analysis using Provena x GS7 and Boyer x GS7 RILs identified multiple loci of interest. Those derived from GS7 were located on chr4A, 5C, and 7A, while loci from Boyer were found on chr1D and 7A, along with two Provena-derived QTL (*QPc_Provena_2A* and *QPc_Provena_7A*). Notably, *QPc_Provena_7A* and *QPc_Boyer_7A* both co-locate with *QCr.cdl11-13A*, a GS7-derived QTL reported by Babiker et al. (2015), which was previously identified in both Provena x GS7 and Boyer x GS7 mapping populations across three trials in the U.S. environment. Another difference between this study and the findings from Babiker et al. (2015) is the detection of *QPc_GS7_4A.1* at 352 Mb in the Provena x GS7 population, a locus previously detected in the Provena × Boyer population in the US, where the resistance allele originated from Boyer. The discrepancy in QTL identification between our study and Babiker et al. (2015) could be attributed to factors such as environmental variation, genotype-environment interactions, QTL epistasis, or differences in pathogen mixtures used at the nurseries during evaluation (Lindhout 2002). To inform the oat community, we screened the developed KASP markers in a collection of 182 oat lines to establish correlations with oat crown rust resistance in the field. We note that although the approach of using a *t*-test to determine correlations is reasonably reliable, it does not account for population structure in the oat population, which can introduce biases to determine true allelic effects. Future genome-wide genotyping of the oat collection will allow us to conduct more in-depth analysis using a mixed-model approach to validate KASP markers.

The strongest loci in this study are *QPc_GS7_4A.2*, detected in the trials MJ23 and MJ24, and *QPc_Boyer_7A*, identified in the CB24 trial. The QTL *QPc_GS7_4A.2* overlapped with an APR QTL on chr4A reported by Nazareno et al. (2022) and a QTL region previously associated with the ASR genes *Pc61* and *Pc64* (Klos et al. 2017). Analysis of the *QPc_GS7_4A.2* haplotype at the QTL across the differential set identified the Pc61 differential line (Coker234) as one of the carriers, but not the Pc64 differential line. In the oat collection, the *QPc_GS7_4A.2* resistance haplotype was also present in other lines postulated to carry *Pc61*, such as Quoll, Wizard, Warrego, and Barcoo. The rust phenotyping experiment at the seedling stage using 20 *Pca* isolates showed similar resistance profiles between GS7 and the Pc61 differential line. The seedling resistance assay and subsequent QTL mapping analysis using 30 RILs carrying contrasting alleles at *QPc_GS7_4A.2* further confirmed the association of this locus with seedling resistance. All these findings, along with Klos et al. (2017) identifying the same genomic region linked to *Pc61*, suggest that the mapped QTL in GS7 is closely linked to the *Pc61* locus or potentially represents *Pc61* itself. Furthermore, based on the distribution of virulent isolates across trials, *QPc_GS7_4A.2* was effective in MJ23 and MJ24, but not in CB24, likely due to the presence of *Pc61*-virulent pathotypes in CB24, which were absent in the MJ23 trial.

In CB24, the resistance in the Provena x GS7 RILs was attributed to the QTL *QPc_GS7_4A.1* and *QPc_Provena_7A*. In the oat collection, the *QPc_GS7_4A.1* locus was significantly associated with resistance in both MJ23 and CB24, suggesting it may be a potential APR locus effective across multiple environments. This locus is collocated with previously identified APR loci identified by Babiker et al. (2015) and Nazareno et al. (2022). The presence of *QPc_GS7_4A.1* and *QPc_GS7_4A.2* in GS7 and their difference in effectiveness across environments suggests that GS7 carries both ASR (likely *Pc61*) and APR. The co-presence of the ASR gene with APR loci has been previously reported. For example, the oat line Garry carries multiple ASR genes (*Pc24*, *Pc25*, *Pc26*) alongside APR genes (*Pc27* and *Pc28*), and the oat line TAM O-301 harbors *Pc58* and additional APR-contributing genes (Upadhyaya and Baker 1960, 1962; Carson 2017; Hoffman et al. 2006). Co-location of APR and seedling resistance genes has also been documented in wheat. For example, *Lr12* (APR) and *Lr31* (ASR) both map to chromosome 4B, and their complementary interaction contributes to resistance (Singh et al. 1999). Similarly, a major field stem rust resistance gene co-locates with *Sr12* in ‘Thatcher’ wheat (Hiebert et al. 2016).

The oat line Coker227 (*Pc60*), which shares a high genetic similarity with Coker234 (*Pc61*) (Nguyen et al. 2024, 2025a) but showed a different seedling resistance profile, also carries the *QPc_GS7_4A.2* resistance haplotype. This is likely a result of GS7’s descent from Pc60 in the pedigree, with both Coker227 (*Pc60*) and Coker234 (*Pc61)* originating from a common source *A*. *sterilis* PI 287211 (Carson 2017). Moreover, the *QPc_GS7_4A.2* resistance haplotype was also found in several crown rust-resistant lines not yet cataloged to carry any known *Pc* gene: TAM-O-405, Drummond, and Mannus. Of these three lines, Mannus has a pedigree connection with Coker234 (*Pc61)*. These lines may potentially harbor the *QPc_GS7_4A.2* as part of their resistance mechanism, though further studies are needed to confirm this. Oat cultivars Volta-2 and Aladdin-1, previously postulated to carry *Pc91* (Nguyen et al. 2025a), were also found to harbor the *QPc_GS7_4A.2* resistance haplotype. Interestingly, a recent genome-wide association study identified a shared genomic interval in the *Pca* genome significantly associated with virulence to both *Pc61* and *Pc91* (Hewitt et al. 2024), suggesting a possible mechanistic link between these resistance loci in oats. Our findings align with this observation, supporting a connection between *Pc61* and *Pc91*.

A candidate gene search identified 25 of 69 annotated genes in the 4A QTL interval that are putatively linked to disease resistance mechanisms. Notably, a cluster of 10 Disease Resistance RGA5 and three *ACD6* genes on chromosome 4A were found in this region. These RGA5 belong to the gene family that encodes NB-LRR proteins in rice, known to mediate resistance to the fungal pathogen *Magnaporthe oryzae,* and exhibit diverse functions in *Avr* recognition (Césari et al. 2014). On the other hand, in Arabidopsis, an *ACD6* was recently identified as the causal gene for leaf senescence (Jasinski et al. 2021). Notably, a gene annotated as Pik2 was identified approximately 2 Mb from the RGA5 cluster within the QTL interval. Pik2, like RGA5, is an NLR protein, and they are functionally related. In rice, RGA5 acts as the sensor in the sensor-helper pair RGA5–RGA4, while Pik2 serves as the helper in the Pik1–Pik2 pair (Zdrzałek et al. 2020). These genetically linked NLR pairs typically operate as sensor-helper systems, where one component recognizes pathogen effectors and the other activates immune responses (Zhai et al. 2011; Zdrzałek et al. 2020). In addition, a putative protein WRK49 was also found. In wheat, WRK49 was claimed to confer differential high-temperature seedling-plant resistance to *Puccinia striiformis* f. sp. *tritici* (*Pst)* (Wang et al. 2017). Furthermore, there is a putative protein Ankyrin repeat-containing protein NPR4 in the QLT region, which was orthologous to *TaANKTM1B-4, TaANKTM2B-1, TaANKTM1D-6, TaANKTM3B-9,* TaANKTM4B-5 in wheat. The ankyrin-transmembrane (ANKTM) subfamily is the most abundant subgroup of the ANK superfamily, with roles in pathogen defense (Hu et al. 2022). A homolog of these genes, *TaANKTM2A-5*, was found to regulate powdery mildew resistance in wheat (Hu et al. 2022). The candidate genes listed here could be potential targets for cloning from the resistant parent to support functional characterisation.

In conclusion, this study emphasises the potential of GS7 and Boyer as a useful source of crown rust resistance in Australia. The KASP markers were developed for *QPc_GS7_4A.1, QPc_GS7_4A.2, QPc_GS7_7A,* and *QPc_Provena_7A/QPc_Boyer_7A, which* will be valuable for marker-assisted breeding in oat improvement programs. It still remains to be investigated if QTL *QPc_GS7_4A.*2 is identifying a gene cluster that contains likely a seedling resistance locus that is closely linked to *Pc61* and potentially *Pc61* itself. Resolving this question will require additional research including genetic tests for allelism, fine mapping and/or TEnSeq pipelines such as MutRenSeq (Mutagenesis and the Resistance gene Enrichment and Sequencing) (Zhang et al. 2020), which could be enabled by the recent advances in oat genomics (Peng et al. 2022; Kamal et al. 2022; Zhang et al. 2025). Future research should focus on stacking these QTL to evaluate their interaction in a common background and the dissection of the mode of action of resistance genes once isolated.

## Supplementary Information

Below is the link to the electronic supplementary material.

Supplementary file1 (XLSX 70 kb)

Supplementary file2 (XLSX 3188 kb)

Supplementary file3 (XLSX 364 kb)

Supplementary file4 (DOCX 2781 kb)

## Data Availability

The DArTSeq genotypes of the oat lines included in this study are deposited in the CSIRO Data Access Portal repository 10.25919/x7sk-qp24. The scripts for genotypic data analysis and QTL mapping are available on GitHub at https://github.com/duongnguyen1987/QTL_mapping.
