# Supplementary material for: QTL mapping of oat crown rust resistance in Australian fields and identification of a seedling resistance locus in oat line GS7
Source: Theor Appl Genet. 2026 Jan 19;139(1):41. doi: 10.1007/s00122-025-05145-x (PMC12815998; doi:10.1007/s00122-025-05145-x)

**QTL mapping of oat crown rust resistance in Australian fields and identification of a seedling resistance locus in oat line GS7.**

**Nguyen et al.**

**Supplementary File 3**

**Fig. S1.** Pairwise LOD score heatmaps for linkage analysis across 21 chromosomes of A the Provena x GS7 RILs with 4,493 SNPs grouped in 43 linkage groups and B the Boyer x GS7 RILs with 5,048 SNPs grouped in 40 linkage groups. Red colour indicates closely linked markers (high LOD score) whereas, blue colour indicates non-linked markers (low LOD score and high recombination fraction). Grid lines divide the binned markers by chromosomes.


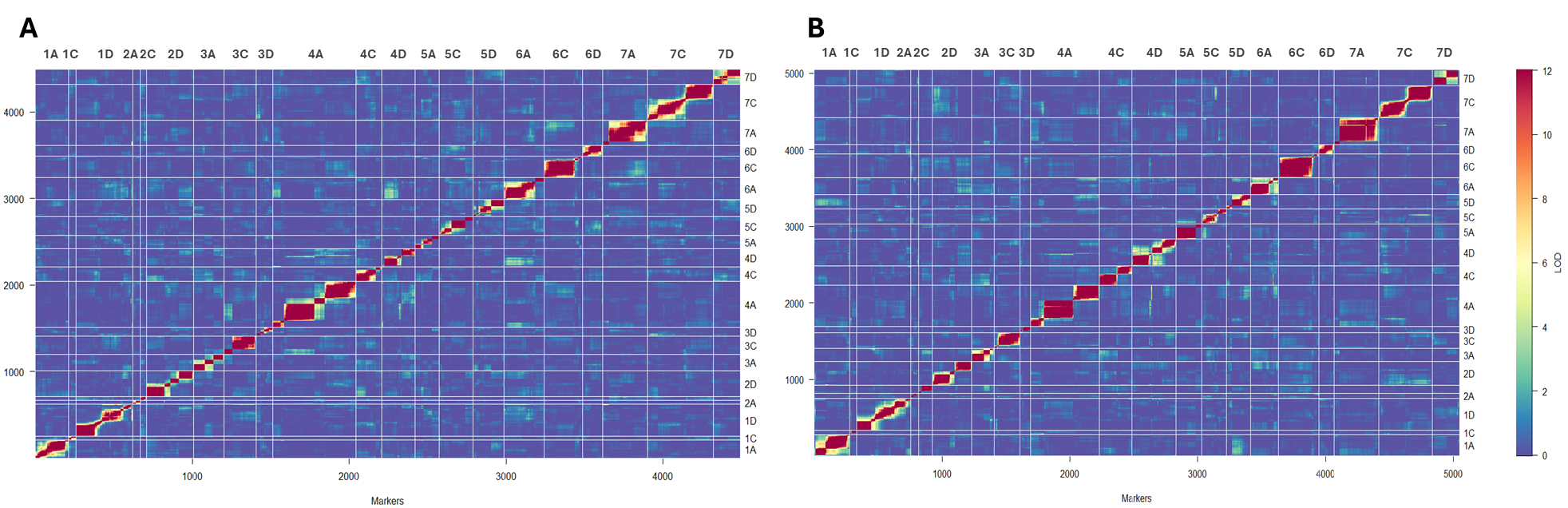


**Fig. S2** Collinearity between the linkage map constructed in this study and the physical map from the reference genome OT3098 v2 for Provena x GS7 RILs. The graphs were constructed with 4493 markers. The “r” value is the correlation coefficient. Blue dots represent matches, while red dots indicate mismatches where markers in the genetic map correspond to different physical chromosomes.


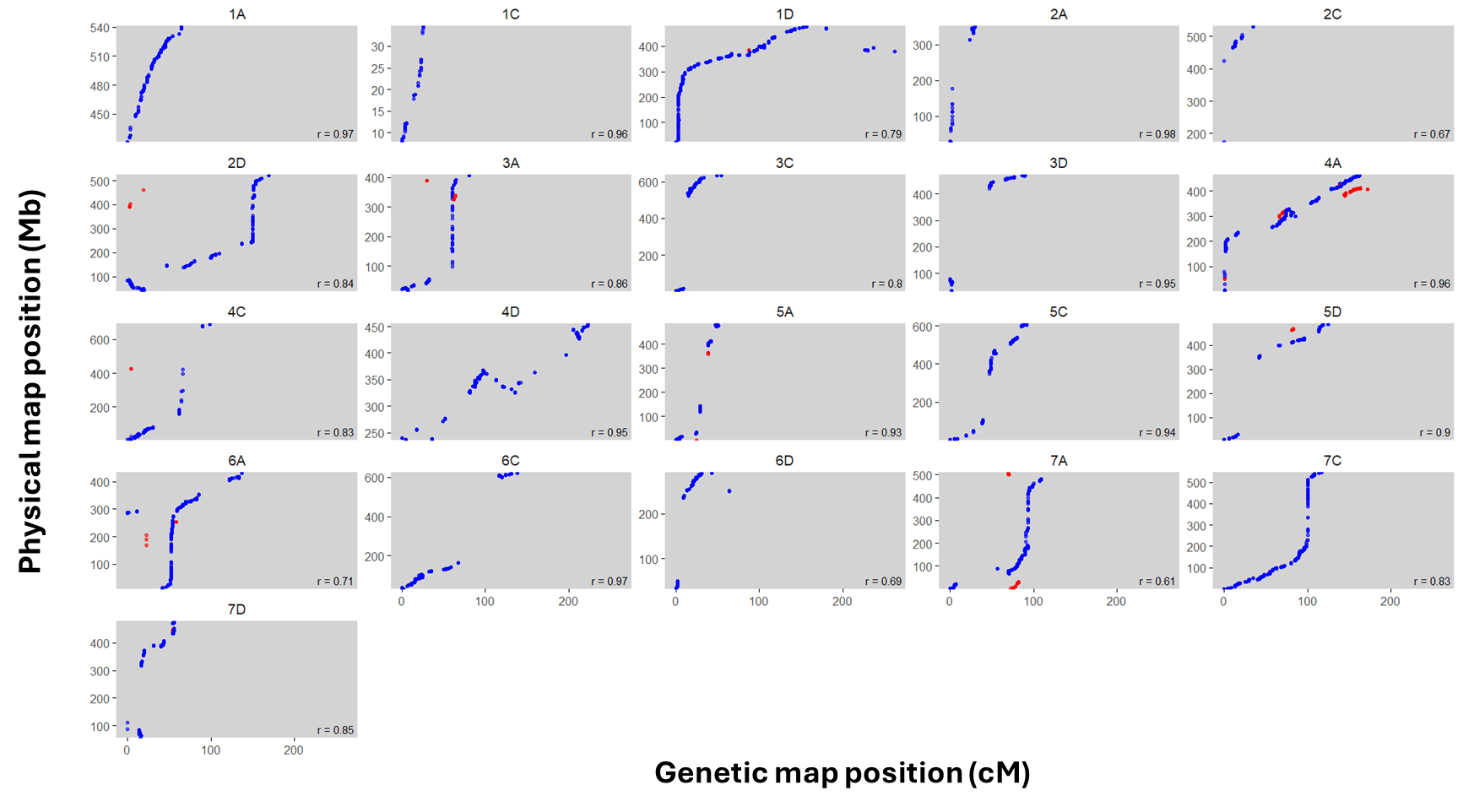


**Fig. S3** Collinearity between the linkage map constructed in this study and the physical map from the reference genome OT3098 v2 for Boyer x GS7 RILs. The graphs were constructed with 5048 markers. The “r” value is the correlation coefficient. Blue dots represent matches, while red dots indicate mismatches where markers in the genetic map correspond to different physical chromosomes.


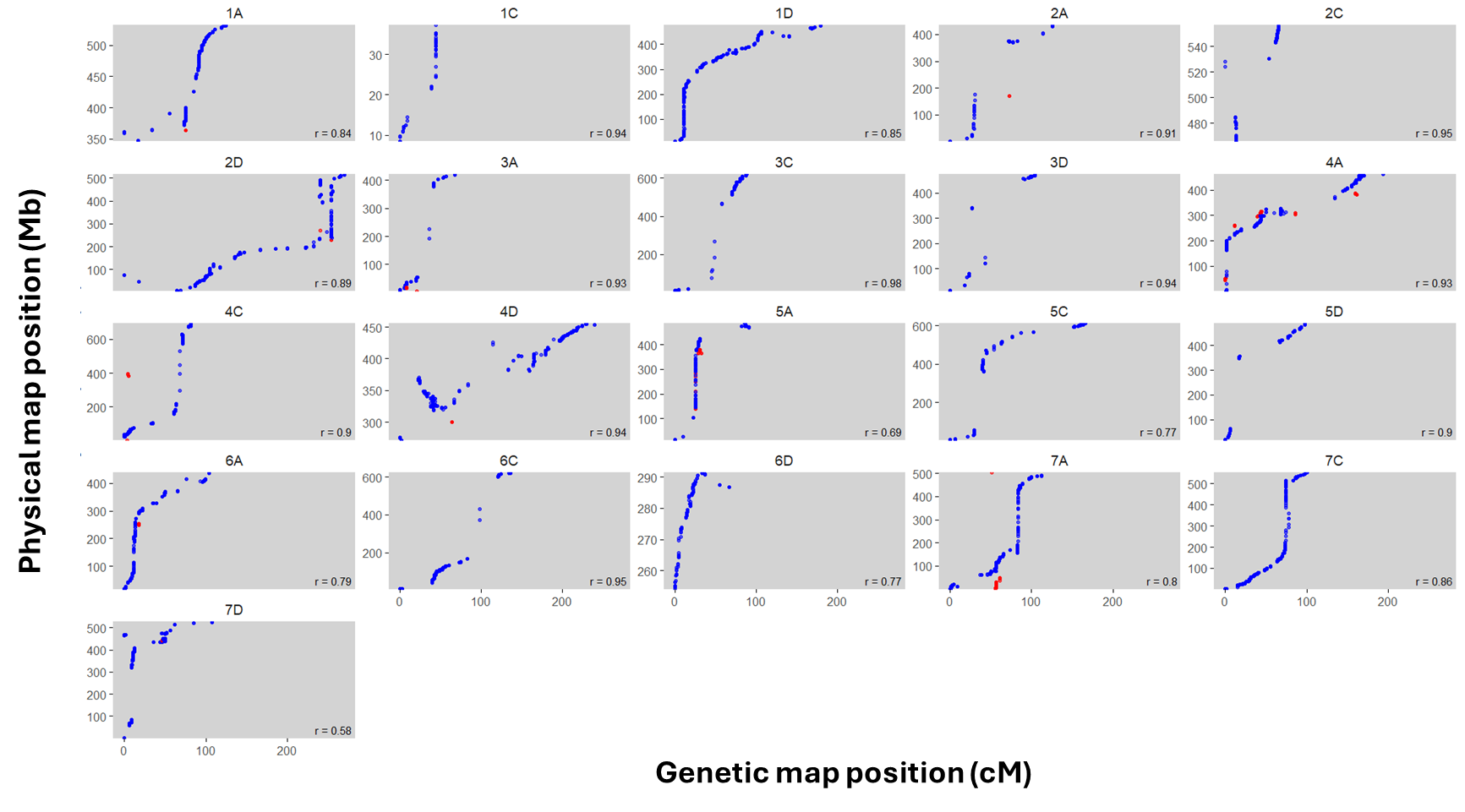


**Fig. S4.** Linkage Disequilibrium (LD) on chromosome 4A and 7A in genetic maps from **A** Provena × GS7 RILs and **B** Boyer × GS7 RILs. Triangles mark the genetic locations of QTL identified on chr4A and chr7A in Provena x GS7 RILs and on chr7A in Boyer x GS7 RILs. The colour gradient represents LD values (R²) ranging from 0 to 1.


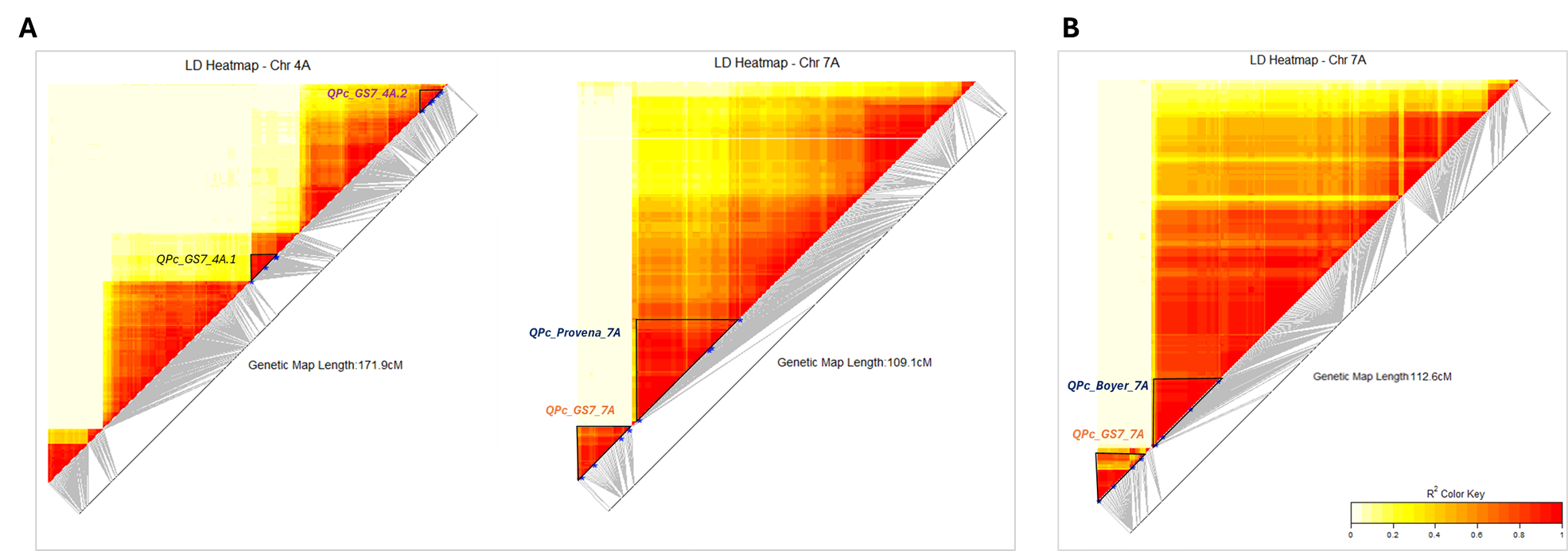


**Fig. S5.** QTL mapping using BLUPs (best linear unbiased predictions) derived from mixed models across environments for Provena x GS7 RILs (left) and Boyer x GS7 RILs (right) . Lod threshold (dashed lines) = 3.


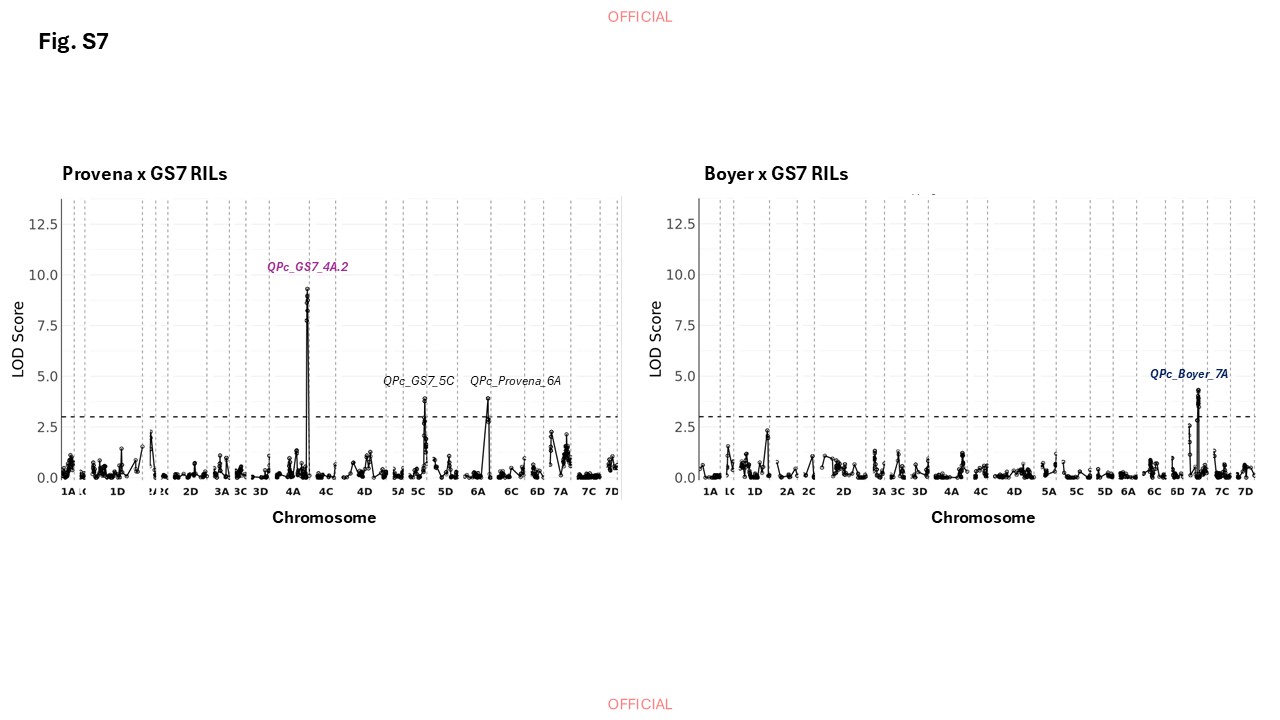


**Fig. S6 A** Confusion matrices showing the proportion of resistant (R) and susceptible (S) lines relative to total observations, based on marker-predicted and observed phenotypes in two field trials, Manjimup 2023 (MJ23) and Cobbitty 2024 (CB24). **B** Heatmaps showing marker performance metrics calculated from confusion matrices, including prediction accuracy, sensitivity, and specificity. Numbers within cells indicate the metric value, while colour intensity reflects relative performance (red = low, blue = high).


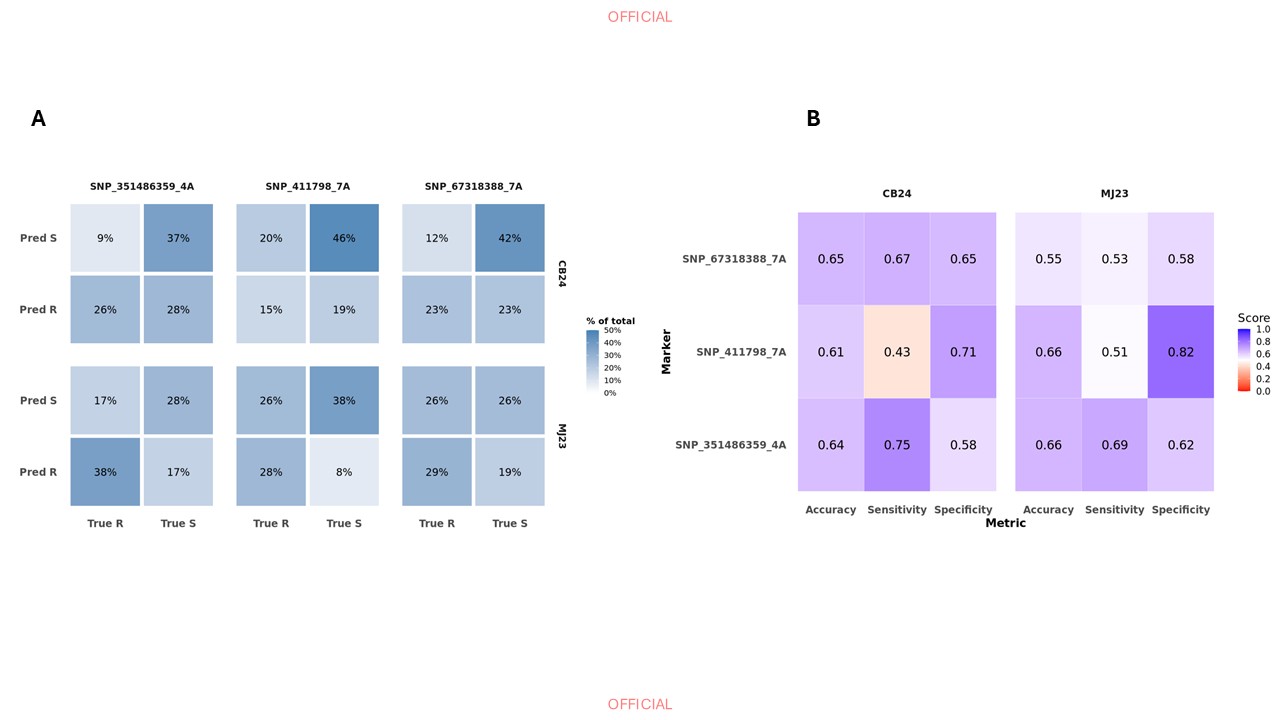


**Fig. S7 A** Comparative mapping of recent QTL studies for oat crown rust resistance significantly associated with genomic regions in chromosomes 4A and 4D. The regions were identified by blasting associated markers to OT3098 v2 reference genome; **B** Syntenic plot comparing 4A and 4D in Oat (OT3098 v2 ref). The red box indicates the genomic region of the QTL QPc.APR/Pc61 in GS7. The black box indicates the duplicated regions in chr4D.


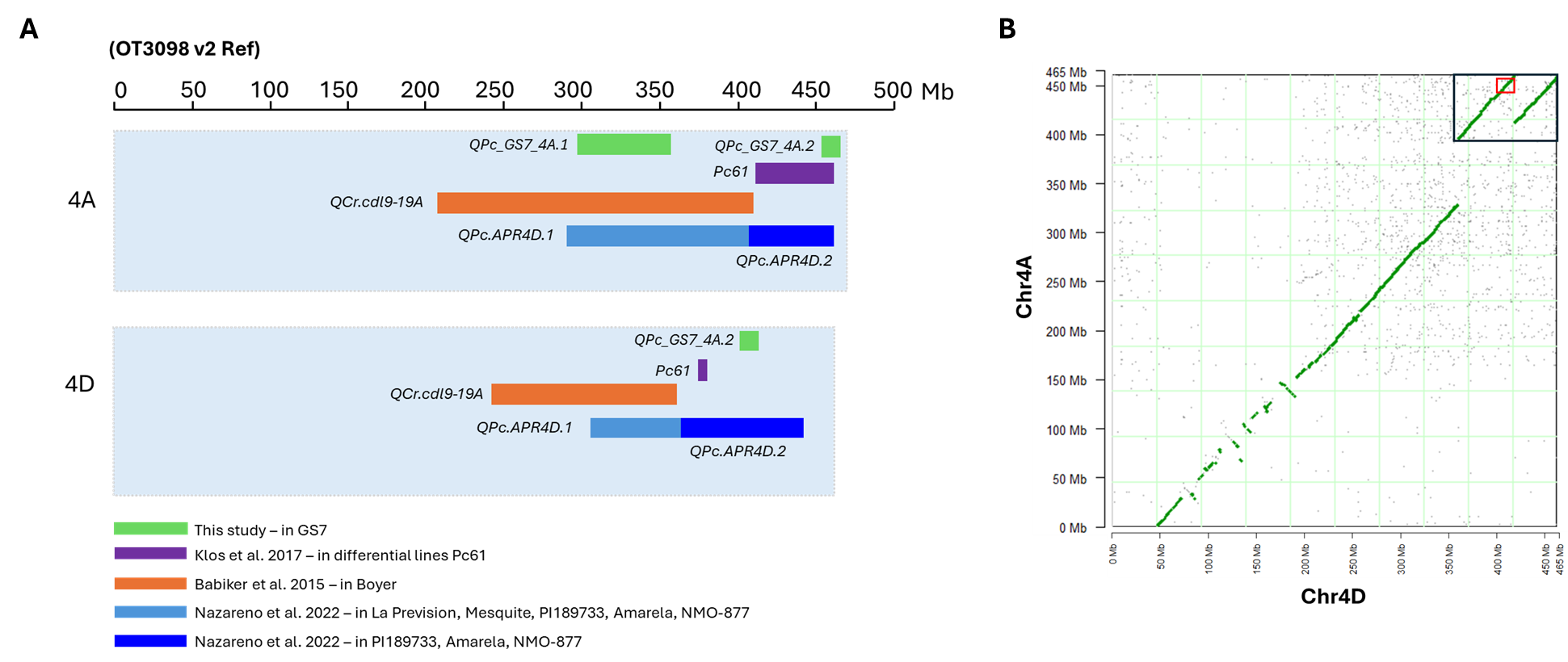


**Fig. S8** QTL mapping of rust score at seedling stage in a subset of Provena x GS7 RILs (n=30) carrying the contrasting allele at the QTL QPc_GS7_4A2 identified from genetic mapping rust resistance in the fields Manjmup 2023 and Manjmup 2024. Lod threshold (dashed lines) = 3.


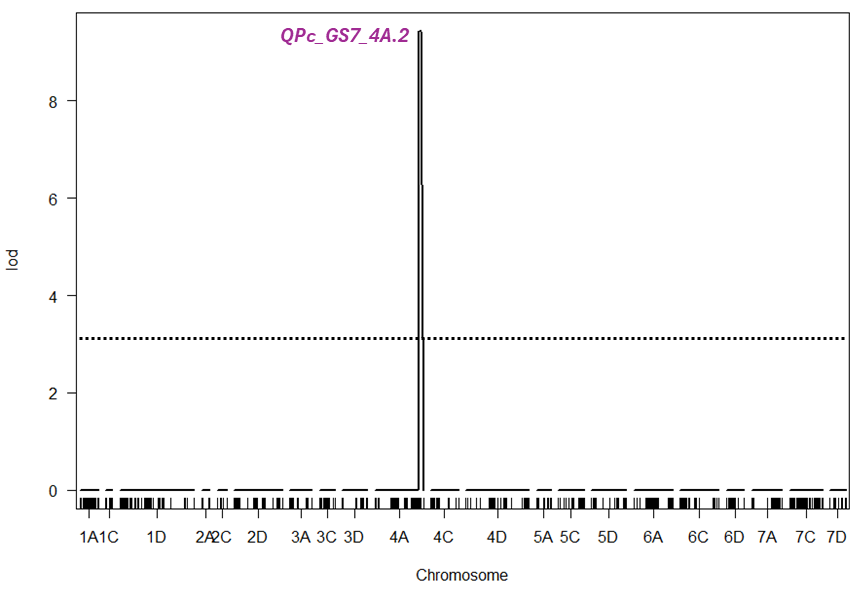

Supplement: Supplementary file 4 — Supplementary file4 (DOCX 2781 kb) [file 122_2025_5145_MOESM4_ESM.docx]
